# Modern Immunotherapy in the Treatment of Triple-Negative Breast Cancer

**DOI:** 10.3390/cancers14163860

**Published:** 2022-08-10

**Authors:** Jakub Wesolowski, Anna Tankiewicz-Kwedlo, Dariusz Pawlak

**Affiliations:** 1 Department of Pharmacodynamics, Faculty of Pharmacy, Medical University in Bialystok, 15-089 Bialystok, Poland; 2 Department of Monitored Pharmacotherapy, Faculty of Pharmacy, Medical University in Bialystok, 15-089 Bialystok, Poland

**Keywords:** triple-negative breast cancer, immunotherapy

## Abstract

**Simple Summary:**

This review summarizes reports from the latest clinical trials assessing the safety and clinical effectiveness of new biological drugs stimulating the immune system to fight cancer. The aim of this study is to show the enormous therapeutic potential of monoclonal antibodies in the treatment of cancer, in particular triple negative breast cancer (TNBC). Introduction of these innovative drugs to the standard clinical cancer therapies, including TNBC, allows for an increase in the response rate to the applied treatment, and consequently extending the lives of patients suffering from cancer. We hope to draw attention to the extremely difficult-to-treat TNBC, as well as the importance of the development of clinical trials evaluating drugs modulating the immune system in TNBC therapy.

**Abstract:**

Triple-Negative Breast Cancer is a subtype of breast cancer characterized by the lack of expression of estrogen receptors, progesterone receptors, as well as human epidermal growth factor receptor 2. This cancer accounts for 15–20% of all breast cancers and is especially common in patients under 40 years of age, as well as with the occurring BRCA1 mutation. Its poor prognosis is reflected in the statistical life expectancy of 8–15 months after diagnosis of metastatic TNBC. So far, the lack of targeted therapy has narrowed therapeutic possibilities to classic chemotherapy. The idea behind the use of humanized monoclonal antibodies, as inhibitors of immunosuppressive checkpoints used by the tumor to escape from immune system control, is to reduce immunotolerance and direct an intensified anti-tumor immune response. An abundance of recent studies has provided numerous pieces of evidence about the safety and clinical benefits of immunotherapy using humanized monoclonal antibodies in the fight against many types of cancer, including TNBC. In particular, phase three clinical trials, such as the IMpassion 130, the KEYNOTE-355 and the KEYNOTE-522 resulted in the approval of immunotherapeutic agents, such as atezolizumab and pembrolizumab by the US Food and Drug Administration in TNBC therapy. This review aims to present the huge potential of immunotherapy using monoclonal antibodies directed against immunosuppressive checkpoints—such as atezolizumab, avelumab, durvalumab, pembrolizumab, nivolumab, cemiplimab, tremelimumab, ipilimumab—in the fight against difficult to treat TNBCs as monotherapy as well as in more advanced combination strategies.

## 1. Introduction

It is estimated that in 2020 there were approximately 2.3 million patients diagnosed with breast cancer (BC) worldwide and 685,000 died of BC [1]. It is supposed that triple negative breast cancer (TNBC) may account for 10–15% of diagnosed breast cancer [2]. This subtype of tumor is often found in patients under 40 years of age and/or with an occurring BRCA1 mutation [3]. Extensive research was conducted to better recognize the molecular phenotypes of breast cancer, which helps adjust treatment and develop new therapeutic opportunities. The clinical classification of BC includes hormone receptor-positive (HR+) tumors with the expression of estrogen (ER) and/or progesterone (PR) receptors, human epidermal receptor 2 (HER2) -enriched tumors with overexpression of HER2 in the absence of HR expression as well as triple-negative tumors without the expression of these three receptors [4]. Furthermore, BC can be categorized into molecular subtypes based on the immunohistochemical markers and complementary DNA (cDNA) microarrays:

- Luminal A (ER and PR positive and HER2 negative);
- Luminal B (ER and PR positive and HER2 positive or negative);
- Basal-like (ER and PR negative, HER2 positive or negative);
- HER2 overexpressing (ER and PR negative and HER2 positive) [5].

As TNBC do not express ER, PR, and HER2, TNBC is more diverse than other types in terms of worse outcomes, narrow therapeutic possibilities, and malignant characteristics such as rapid growth and formation of metastases [6]. Its aggressive characteristics reflect the dismal prognosis: the median overall survival of metastatic TNBC is 8–15 months [7]. Because of the actionable molecular targets, chemotherapy remains the main option in TNBC treatment. However, systemic chemotherapy provokes adverse effects and most patients quickly develop resistance. Furthermore, TNBC tends to relapse frequently, which creates the necessity to develop new therapeutic strategies [8].

## 2. Role of the Immune System

The immune system is a very important factor in the fight against cancer. Its role is to prevent the growth of neoplasm by destroying cancer cells as well as decreasing the possibility to metastasize. However, it can promote tumor progression [9]. The immune system is under self-control by immune checkpoints to protect the body’s natural, healthy cells from immune-mediated death, a process called peripheral tolerance [10]. The basis of this process is the recognition and binding of a T-cell receptor (TCR) to an antigen presented in the major histocompatibility complex (MHC) on the surface of an antigen-presented cell (APC). The cytotoxic T-lymphocyte-associated antigen-4 (CTLA-4), programmed cell death-1 (PD-1), and its ligands (PD-L1) are involved in the suppression of the T-cell immune response. It is supposed that the CTLA-4 and PD-1 pathways play roles at different stages of immune system activity. CTLA-4 is responsible for inhibiting a potentially autoaggressive T-cell at the early stages, especially in the lymph nodes. While the PD-1 pathway provides self-tolerance by regulating already activated T-cells at the later stages of an immune response—mostly in peripheral tissue [11].

Immunotherapy has shown high efficacy in the treatment of some types of tumors—such as melanoma, kidney and non-small cell lung cancer (NSCLC)—including immune checkpoint inhibitors (ICIs) as indications for treatment [12,13,14]. In TNBC with a lack of ER, PR and HER2, the use of monoclonal antibodies alone or with other therapeutic options such as chemotherapy, radiotherapy as well as select targeted therapies seems hopeful in early or advanced stages of TNBC. In this review, we aim to shed a light on immunotherapeutic opportunities using monoclonal antibodies approved by the U.S. Food and Drug Administration (FDA) in the treatment of TNBCs, such as atezolizumab and pembrolizumab, We also discuss additional immune checkpoint inhibitors (ICIs), such as nivolumab, avelumab, durvalumab, cemiplimab, tremelimumab and ipilimumab—currently undergoing clinical trials for evaluation of their safety and efficacy in patients with TNBC.

## 3. CTLA-4 Pathway

CTLA-4 is an immune checkpoint receptor expressed on the surface of activated T-cells. It is a CD28 homolog, however, it binds significantly stronger to B7-1 (CD80) or B7-2 (CD86) molecules presented on the APC [15]. Activation of the T-cells is possible due to the interaction of CD28 with B7-1/2. This leads to the proliferation of T-cells, increased T-cell survival and differentiation due to interleukin-2 production (as a growth factor), as well as increased energy metabolism and upregulation of cell survival genes. Nevertheless, the effect of interaction between CTLA-4 and B7 does not provide stimulation and it is suggested, that suppressive signals may be the result of this process [16]. This is particularly important when the immune system is overactive. Thus, the binding ratio of CD28 and B7 to CTLA-4 and B7 determines whether the T-cells will be activated or inhibited [17]. Furthermore, CTLA-4 may contribute to immune system control through other mechanisms. The expression of CTLA-4 on the surface of regulatory T-cells (Tregs) is constant. Tregs are responsible for regulating the activity of the effector T-cells by downregulating B7 ligands on APCs, leading to limited CD28 costimulation [18]. As a result, of the impairment of CD28:B7-mediated costimulation, there is a limitation of T-cell proliferation and their effector functions [19].

## 4. PD-1/PD-L1 Pathway

Programmed cell death protein 1 (PD-1) is a transmembrane protein from the B7-CD28 family and is referred to as an inhibitory immune checkpoint that affects T-cell regulation. The basis of this process is the interaction of PD-1 with its ligand—PD-L1 or PD-L2. It is thought that the PD-1 pathway contributes to the inhibition of T-cells proliferation, decreased production of interferon-γ (IFN-γ), tumor necrosis factor-α (TNF-α) and interleukine-2 (IL-2), as well as reduced T-cell survival [20].

PD-1 expression is noticed during T-cell activation and is present on the surface of all subsets of T-cells as well as other immune-related cells—such as B cells, natural killer (NK) cells, and myeloid cells [21]. Interestingly, PD-1 is expressed significantly higher on tumor-infiltrating T-cells [22]. It has been shown that higher expression of PD-1 is related to exhausted T-cells leading to an impaired antitumor response [23].

Binding T-cells by their TCR to an antigen expressed on the cell surface, leads to T-cell activation, proliferation, PD-1 upregulation, and inflammatory cytokine production. Their secretion may contribute to PD-L1 expression on the cancer cell surface, and subsequently after PD-1 and PD-L1 binding, results in suppression of TCR-mediated immune response [24]. Sustained expression of PD-1 and its ligands is usually noticeable during certain conditions—such as chronic infections or cancer. Thus, improvement of T-cell functions and reduction of tumor burden may be achieved by blocking the PD-1 pathway [25,26].

Moreover, it is thought that vascular endothelial growth factor A (VEGF-A)—a proangiogenic molecule produced from cancer cells—is a meaningful factor in the development of an immunosuppressive microenvironment. The basis of this process is the accumulation of myeloid-derived suppressor cells, the inhibition of dendritic cell maturation as well as the induction of regulatory T-cells. A recent study demonstrated that VEGF-A produced in the tumor microenvironment increased the expression of PD-1 and other inhibitory checkpoints involved in CD8+ T-cell exhaustion, which could be counteracted by antiangiogenic agents targeting VEGF-A-VEGFR [27].

## 5. Immunotherapy Using PD-1/PD-L1 Checkpoint Inhibitors

Cancer cells very often acquire the features that enable them to evade the immune system’s responses through immune checkpoint pathways or by forming an immunosuppressive microenvironment that causes tumor recognition impairment and disease development [28].

**Figure 1:**
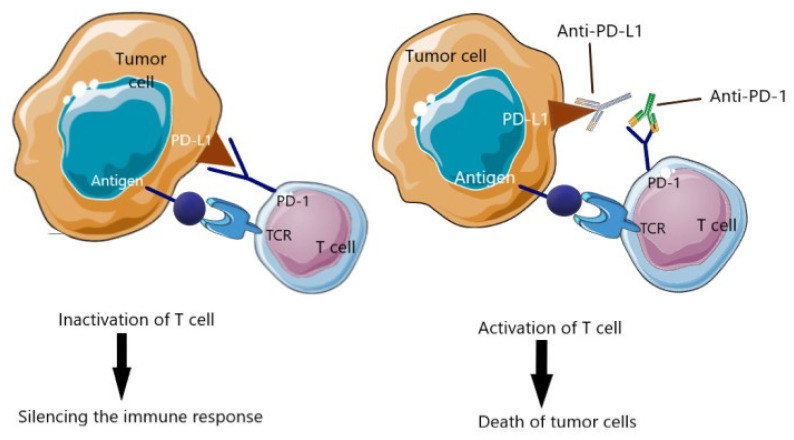
Immune checkpoint inhibitors. The expression of PD-L1 allows tumor cells to switch T-cells off and keep the cancer cells from being killed. The use of immune checkpoint inhibitors permits boosting the immune response to kill the cancer cells. TCR, T-cell receptor; PD-1, programmed death protein 1; PD-L1, programmed death-ligand 1 [Own illustration designed on the basis of [4]].

The immune checkpoints pathway is essential to protect normal tissue from immune system hyperactivity. The expression of PD-L1 in TNBC is about 20%, which is correlated with the development of resistance to specific CD8+ T-cells and higher tumorigenesis [29]. T-cells, B cells, natural killer (NK) T-cells express PD-1 receptor on their surface and it is correlated with the silencing of the immune system by the tumor. The purpose of using ICIs is to elicit an immune response by suppressing the inhibitory pathways (Figure 1) [30]. Thus, these features suggest a promising therapeutic target for this disease.

**Figure 2:**
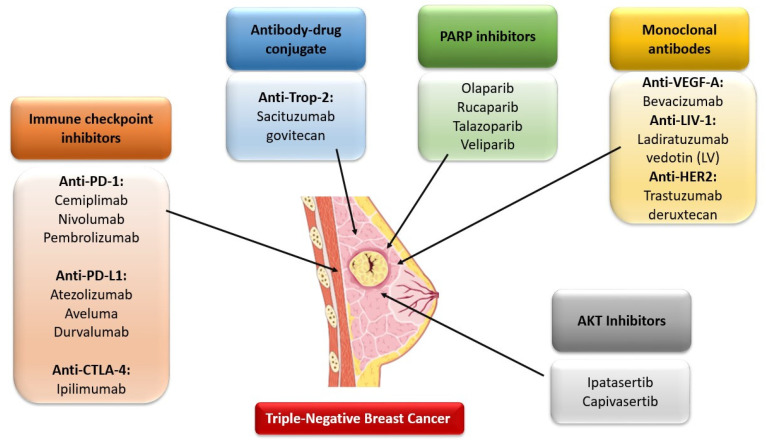
Drugs including immunotherapeutic agents that are currently being investigated in TNBC treatment [Own illustration designed on the basis of [8]].

Drugs including immunotherapeutic agents that are approved for TNBC therapy such as atezolizumab and pembrolizumab as well as other drugs such as AKT inhibitors, monoclonal antibodies, Poly ADP-Ribose Polymerase (PARP) inhibitors and antibody-drug conjugate currently being investigated in TNBC treatment are shown in Figure 2.

### 5.1. Atezolizumab

Atezolizumab is an Fc-engineered, humanized, monoclonal IgG1 antibody that selectively binds to programmed death-ligand 1 (PD-L1) and inhibits its interactions with PD-1 and B7.1 receptors causing T-cells more sensitive to the tumor. In a phase one of the clinical trial, atezolizumab as a single agent was evaluated in terms of safety and antitumor activity and showed good clinical outcomes and potential benefits in patients with metastatic TNBC especially in earlier lines of treatment or those with higher levels of tumor-infiltrating immune cells (ICs) and PD-L1 positive ICs [31].

The IMpassion130 was carried out to investigate the antitumor effects and clinical benefits of combining atezolizumab with taxane chemotherapeutic agent—paclitaxel in the form of nanoparticle albumin-bound (nab-paclitaxel)—in patients with unresectable, locally advanced or metastatic TNBC. In the first interim analysis, this combined therapy increased progression-free survival (PFS) compared with placebo plus nab-paclitaxel—(7.2 months compared to 5.5 months, respectively). Furthermore, higher differences were noticed among patients with PD-L1-positive tumors. The median PFS was 7.5 months in the atezolizumab group and 5.0 months in the placebo groups. Interestingly, therapy with atezolizumab prolonged overall survival (OS), particularly in groups with PD-L1 expression. There were 3.7 months of difference between the atezolizumab and control groups (21.3 vs. 17.6 months). In addition, there was a ten-month difference in median overall survival between the PD-L1 groups receiving atezolizumab and placebo (25.0 vs. 15.5 months properly). There were no significant differences between races. The most meaningful benefits occurred in the oldest patient TNBC group (≥65 years of age) [32].

The next interim overall survival analysis of IMpassion130 confirmed the previous results of improvement in PFS and OS. Thus, these findings clearly indicate antitumor activity as well as clinical benefits of combination therapy with atezolizumab and nab-paclitaxel, particularly for patients with PD-L1 immune cell-positive metastatic TNBC [33]. Based on the findings from the IMpassion130 clinical trial, the U.S. Food and Drug Administration and the European Commission have approved atezolizumab plus nab-paclitaxel for the treatment of adult patients with unresectable locally advanced or metastatic TNBC whose tumors have PD-L1 expression ≥1% and who have not received prior chemotherapy for metastatic disease [34].

The safety and efficacy of therapy with atezolizumab and nab-paclitaxel were also evaluated in a Japanese subgroup. The outcomes were consistent with those received from IMpassion130. This therapy prolonged PFS and showed a high objective response rate (ORR) even higher than the ORR announced in the IMpassion130 trial. Objectively, atezolizumab had a demonstrated safety profile and was well-tolerated in the Japanese population however, there were adverse events such as alopecia, peripheral sensory neuropathy, decreased neutrophil count, nasopharyngitis and decreased white blood cell count occurring more frequently than in the overall Impasssion130 population. These findings were limited by the small study population but can be contributed to the adjustment of combined therapy with the use of atezolizumab and nab-paclitaxel to the standards of treatment available in Japan [35].

### 5.2. Pembrolizumab

Pembrolizumab is a humanized, monoclonal IgG4 antibody that selectively binds to programmed death-1 (PD-1) receptor and inhibits interaction with its ligands—PD-L1 and PD-L2. The KEYNOTE-012 was the first study evaluating the safety and anti-cancer efficacy of pembrolizumab as a monotherapy in 32 enrolled, previously treated with chemotherapy patients with PD-L1-positive, advanced TNBC. In this 1b cohort study, this single agent showed an encouraging overall response rate (ORR) of 18.5% and with a demonstrated safety profile consistent with previous findings [36]. However, in the phase two of the KEYNOTE-086 study (cohort A) pembrolizumab did not provide significant outcomes in patients with mTNBC. ORR was 5.3% in a total group of 170 previously treated PD-L1-unselected patients [37]. Interestingly, in cohort B of the same investigation, ORR was 21.4% in patients with untreated PD-L1-positive tumors. Moreover, pembrolizumab demonstrated a clinically relevant median duration of response of 10.4 months and was well-tolerated. Thus, these results indicate that pembrolizumab has greater efficacy in the treatment of earlier metastatic stages [38].

In addition, some results point to the immunomodulatory potential of chemotherapy to improve anticancer response to ICIs [39]. A clinically significant antitumor effect of pembrolizumab combined with chemotherapy was demonstrated in the KEYNOTE-355 phase three clinical trial. In this randomized clinical trial—with 1372 patients enrolled—the combined therapy with this monoclonal antibody prolonged PFS as much as 4.1 months compared with placebo plus chemotherapy (9.7 months in the pembrolizumab plus chemotherapy group and 5.6 months in the chemotherapy alone group) among mTNBC patients with PD-L1 expression in whom a combined positive score (CPS) was ≥10. Furthermore, this trial included not only one chemotherapeutic option, such as presented in the IMPASSION-130 clinical trial (atezolizumab and nab-paclitaxel), but investigated several therapeutic standards—such as nab-paclitaxel, paclitaxel, or gemcitabine plus carboplatin—expanding the spectrum of possibilities. There were no new safety concerns. Adverse effects were noticed in 68% of the pembrolizumab group and 67% of the placebo group [40].

#### Pembrolizumab and Radiotherapy

Consistent with the previously noted findings concerning the immunomodulatory effects of radiotherapy (RT) in combination with ICIs, based on increasing cytotoxic T-cell levels and activation of apoptosis pathways, a phase two clinical trial demonstrated promising clinical benefits from the combination of RT and pembrolizumab. This study included a small group of 17 TNBC patients showing poor prognosis. The women were not divided into groups in terms of PD-L1 expression. The median ORR was 17.6%, similar to the findings with pembrolizumab as a single agent in pretreated patients with PD-L1-positive mTNBC. Furthermore, this combined therapy seemed to be safe with-low grade adverse effects. This study was limited to a small single-arm group and more future clinical trials are needed to confirm the clinical effectiveness [41].

### 5.3. Immunotherapy Combined with Antiangiogenic Factor

The impaired function of the tumor blood vessels may contribute to the decreasing levels of cytotoxic T-cells (involved in the fight against cancer) and increasing levels of myeloid-derived suppressor cells (MDSC) and regulatory T-cells (involved in inhibiting of the immune response). In addition, some research indicates that antiangiogenic therapy leads to the augmentation of PD-L1 expression as well as CD8+ T-cell infiltration in the tumor microenvironment. Thus, a reduction of the immunosuppression, as well as sensitizing the tumor to the immune response could be achieved by restoring proper perfusion [42].

Consistent with these promising findings, camrelizumab (PD-1 inhibitor) in combination with apatinib (tyrosine kinase inhibitor, which selectively inhibits vascular endothelial growth factor receptor 2 (VEGFR2)) was was investigated in patients with advanced TNBC. In this two phase clinical trial, this therapy showed a significant ORR of 43.3% and was well-tolerated, irrespective of PD-L1 expression. However, this open-label study had several limitations and large clinical trials with randomization are needed to confirm these results as to compare ICIs in combination with VEGFR2 inhibitors with ICIs plus placebo [7].

### 5.4. Durvalumab with Neoadjuvant Therapy

As previously mentioned, ICIs showed promising therapeutic benefits, especially in combination with chemotherapy. In the GeparNuevo phase two clinical trial, the combination of durvalumab (PD-1 inhibitor) with standard neoadjuvant chemotherapy was evaluated in early TNBC. In this study, the patients were randomized in terms of stromal tumor-infiltrating lymphocytes. Two weeks before starting treatment with chemotherapy, the patients received durvalumab or a placebo in monotherapy. Subsequently, the patients were treated with durvalumab or placebo (every four weeks) and nab-paclitaxel (weekly) for 12 weeks, followed by durvalumab or placebo and epirubicin/cyclo- phosphamide (EC) (four cycles). The use of durvalumab in combined therapy with nab-paclitaxel and EC demonstrated a higher pCR rate than the placebo group (53.4% vs. 44.2% respectively), however without statistical significance (*p* = 0.287). Nevertheless, the patients who had received durvalumab as a monotherapy two weeks before the main treatment showed a higher pCR rate than the placebo group (61.0% vs. 41.4%, *p* = 0.035). Thus, these findings suggest the potential benefits derived from using durvalumab with anthracycline/taxane-based therapy, particularly in patients pretreated with durvalumab as a single agent before the main therapy [43].

### 5.5. Nivolumab

Nivolumab is a human monoclonal antibody directed against the PD-1 immunoregulatory receptor located on the surface of T-cells. Despite the numerous indications for solid tumor treatment (melanoma, non-small cell lung cancer, malignant pleural mesothelioma, renal cell carcinoma, squamous cell cancer of the head and neck, urothelial cancer, colorectal cancer, oesophageal squamous cell carcinoma), research concerning the use of nivolumab in TNBC therapy is limited.

Recently, in the first stage of phase two TONIC clinical trial, nivolumab had shown high clinical efficacy among patients with metastatic TNBC. In this trial, 67 patients were divided into five cohorts, the first one was a control group with a two-week waiting period, and the other four included patients who received the induction treatment consisting of irradiation, low-dose cyclophosphamide, cisplatin, or doxorubicin for two weeks. After this short-term treatment, the therapy was continued for eight weeks with three cycles of nivolumab. In all cohorts, the objective response rate was 20% after full treatment. Additionally, the median progression-free survival was 1.9 months. The best results were noticed in the cisplatin and doxorubicin cohorts, where the ORR was 23% and 35%, respectively. Furthermore, treatment with cisplatin and doxorubicin was associated with the upregulation of immune-related genes involved in the PD-1/PD-L1 and T-cell cytotoxicity pathways. Thus, consistent with these findings pretreatment with the use of immunomodulating chemotherapeutic agents—such as cisplatin and doxorubicin—promotes a more favorable tumor microenvironment and provides a high and durable response to nivolumab, as well as the clinical benefits derived from this induction strategies therapy [44].

### 5.6. Avelumab

Avelumab is another immune checkpoint inhibitor. This human IgG1 monoclonal antibody directed against PD-L1 blocks the PD-1/PD-L1 pathway but does not affect PD-1/PD-L2 interaction [45]. The specific feature that distinguishes this agent is its second mechanism of action. In the preclinical studies, avelumab showed an additional advantage consisting of the ability to destroy human cancer cells by inducing antibody-dependent cell-mediated cytotoxicity (ADCC) [46]. Furthermore, an in vitro study was carried out to evaluate the avelumab-mediated ADCC on TNBC line cells, whose PD-L1 expression was changed with the use of peripheral blood mononuclear cells (PBMC) or purified NK cells from healthy donors. In this research, avelumab showed antitumor activity against TNBC cells independent of the PD-1/PD-L1 pathway. Avelumab increased TNBC cell death through an NK-mediated cytotoxicity mechanism. Additionally, higher expression of PD-L1 on tumor cells was related to increasing sensitivity to avelumab-mediated ADCC. Interestingly, the addition of IL-2 and IL-15 to the tumor cells contributed to the enhancement of the lysis of tumor cells through increased NK cell activity [47]. However, in clinical conditions in patients with metastatic breast cancer, avelumab in monotherapy did not show significant results. The phase one clinical trial JAVELIN enrolled 168 patients with various subtypes of breast cancer. This population included 58 patients with TNBC. The pretreatment of all patients had been a median of three prior therapies for metastatic disease. Among patients with TNBC, 50% of them had been previously treated with ≥2 prior lines of therapy for metastatic disease. The overall objective response rate was 3.0%, and 5.2% in the subgroup with TNBC. Furthermore, the percentage of patients in the overall population with PD-L1-positive tumors achieved a much higher ORR relative to the patients without PD-L1 expression (16.7% vs. 1.6% respectively and 22.2% vs. 2.6% respectively, in the TNBC subgroup). Additionally, avelumab as a single agent was well-tolerated and showed a safety profile consistent with previous findings [48].

### 5.7. Cemiplimab

Cemiplimab is an IgG4 monoclonal antibody blocking the PD-1 pathway. In a clinical trial among patients with metastatic cutaneous squamous cell carcinoma (CSCC), Cemiplimab showed a safety profile and high efficacy. Consistent with these findings, this agent was approved by the U.S. FDA for patients with metastatic CSCC or locally advanced CSCC [49]. However, to date, studies evaluating the safety and efficacy of TNBC therapy are ongoing and results are not yet available.

### 5.8. Tremelimumab

Tremelimumab is one of the first CTLA-4-pathway immune checkpoint inhibitors. This IgG2 monoclonal antibody has antitumor activity potential by inhibiting the CTLA-4 receptor expressed on activated T-cells, preventing the interaction between the antigen-presenting cell ligands B7-1 (CD80), B7-2 (CD86), and CTLA4. Disabling the CTLA-4-mediated inhibition pathway leads to binding the CD28 protein presented on T-cells with its B7-1/2 ligand on APCs. Thus, the use of this agent allows for increasing the activation of T-cells, as well as their effector functions to kill the cancer cells [50,51]. Currently, its safety and antitumor activity have been evaluated in many types of cancer. To date, tremelimumab has no indications for the treatment of any type of breast cancer. Its safety and activity were assessed in patients with HR-positive metastatic BC in combination with a steroidal aromatase inhibitor—exemestane. In this clinical trial, tremelimumab in combined therapy was well-tolerated with stable responses for ≥12 weeks in 42% of patients. Additionally, the immunomodulatory activity of tremelimumab was observed and associated with the augmented expression of inducible costimulator (ICOS) on CD4+ and CD8+ T-cells as well as a decreased number of FoxP3+ Treg cells [51,52]. Furthermore, a phase one clinical trial was conducted to investigate the safety and efficacy of tremelimumab with radiotherapy in patients with metastatic breast cancer. This study enrolled six patients—five with HR+ metastatic BC and one with TNBC. Tremelimumab in this strategy was safe and well-tolerated with the frequent adverse events such as lymphopenia, fatigue and rash. Objective responses were not observed. The most meaningful response was stable disease. Progression-free survival was 1.5 months, and overall survival was 50.8 months [52,53].

### 5.9. Ipilimumab

Ipilimumab is the second CTLA-4-pathway antagonist. It is a human IgG1 monoclonal antibody selectively binding with CTLA-4 receptor presented on the activated T-lymphocytes. To date, ipilimumab is approved by the U.S. Food and Drug Administration for the treatment of melanoma, renal cell carcinoma, colorectal cancer, hepatocellular carcinoma, non-small cell lung cancer and malignant pleural mesothelioma [53,54]. Despite many clinical trials in the treatment of solid tumors, studies focused on the efficacy and toxicity of ipilimumab in the treatment of breast cancer, especially in TNBC, are less abundant. An in vitro study showed that the inhibition of CTLA-4 receptors by ipilimumab has an impact on the release of IL-2 by tumor cells simultaneously regulating the tumor microenvironment and increasing the immune response [55]. In a clinical study in a group of 29 patients with relapsed malignancy, after a single infusion of ipilimumab, enhanced intracellular CTLA-4 expression (probably due to T-cell activation) as well as increased levels of activated T lymphocytes were observed without significant changes in the regulatory T-cell population [56]. In addition, ipilimumab was evaluated in a pilot study in patients with breast cancer. Nineteen patients were enrolled in this clinical trial, 12 of them received ipilimumab—six patients received ipilimumab in monotherapy and six patients received ipilimumab with cryoablation. After the use of combination immunotherapy, an increase in T helper type 1 cytokines, ICOS+Ki67+CD4+, and ICOS+Ki67+CD8+ T-cells was observed as well as an increased CD8+ Tcell/FoxP3+ Treg ratio within the tumor. Thus, consistent with these findings, the combination of Ipilimumab with cryoablation may contribute to the anti-tumor benefits derived from this strategy [57,58].

## 6. Biomarkers

Because not all patients respond to the administered immunotherapy, there is a strong need to identify prognostic and predictive biomarkers of response to the applied treatment, which would allow the selection of patients only to the group that would respond to the applied treatment. As it turns out, the implementation of ICIs for mTNBC therapy in patients with high tumor-infiltrating lymphocytes (TILs) shows encouraging results, which indicates the potential benefits of the applied immunotherapy for these patients [59]. Some authors, for instance, Xiao, Y., et al., in their studies, suggest the use of TILs and immune checkpoint molecules as biomarkers providing information about the effectiveness of treatment using ICIs in patients with TNBC [60]. Interestingly, the selection based on PD-L1 is quite controversial due to the lack of a consistent assessment standard. Additionally, new biomarkers like immune gene signatures liquid biopsy, and gut microbiome are still under research and cannot yet be used in clinical practice. Therefore, it is necessary to develop complex methods and further research into new and already known potential biomarkers of the response to immunotherapy in patients with TNBC [59].

## 7. Immune-Related Adverse Events (IRAEs)

The activation of the immune system using ICIs is also associated with the occurrence of characteristic side effects that differ from chemotherapy due to the mechanism of immune-related toxicity, a delayed onset and prolonged duration. IRAEs usually develop within weeks to months of starting immunotherapy. Nevertheless, they can appear at any time, even after the use of ICIs has been discontinued. The most common IRAEs include dermatologic toxicities, endocrine toxicities, gastrointestinal toxicities and hepatitis. Rarely developing IRAEs involve the cardiovascular, pulmonary, musculoskeletal, ocular, and central nervous systems. Nevertheless, these toxicities can develop in almost any organ. Reducing IRAEs very often requires a comprehensive approach and the involvement of a multidisciplinary medical team. Corticosteroids are most commonly used to mitigate the effects of ICIs, but other measures may also be required, such as additional immunosuppression, delay, or discontinuation of treatment. Therefore, it is also extremely important to educate both healthcare professionals and patients to identify IRAEs early and manage treatment appropriately [61].

## 8. Conclusions

Immunotherapy is without a doubt a groundbreaking achievement having a big impact on the improvement of clinical conditions and could be a life-changing opportunity for patients struggling with a highly aggressive type of breast cancer. Immune checkpoints are the most often used by cancer to switch T-cells off and keep the cancer cells from being killed. The use of immune checkpoint inhibitors permits boosting the immune response to kill the cancer cells. Current studies clearly indicate the necessity to combine the ICIs in various therapeutic regimens in order to increase their effectiveness and clinical benefits (Table 1, Table 2, Table 3, Table 4, Table 5, Table 6, Table 7 and Table 8). Furthermore, there is a strong need to implement immunotherapy in the early-stage setting of metastatic treatment to increase the overall response rate [58].

## Figures and Tables

**Table 1:** Atezolizumab.

NCT Number	Status	Co-Treatment/Intervention	Phase	Participants	Results/Conclusions	References
NCT03125902	Active, not recruiting	Paclitaxel, Placebo	III	651	Combining atezolizumab with paclitaxel did not improve PFS or OS versus paclitaxel alone	[62]
NCT04148911	Recruiting	Nab-Paclitaxel	III	180	Estimated Primary Completion Date: 29 October 2024	[63]
NCT02425891	Completed	Nab-Paclitaxel, Placebo	III	902	Atezolizumab plus nab-paclitaxel prolonged progression-free survival among patients with metastatic triple-negative breast cancer in both the intention-to-treat population and the PD-L1-positive subgroup.	[32,64,65]
NCT03281954	Active, not recruiting	Neoadjuvant chemotherapy with Atezolizumab or Placebo followed by adjuvant continuation of atezolizumab or placebo	III	1520	Estimated Primary Completion Date: 31 December 2023	[66]
NCT03164993	Recruiting	Chemotherapy (pegylated liposomal doxorubicin + cyclophosphamide), Placebo for atezolizumab	II	75	Estimated Primary Completion Date: 31 December 2023	[67]
NCT04690855	Recruiting	Talazoparib, Radiation	II	23	Estimated Primary Completion Date: June 2022	[68]
NCT04584112	Recruiting	Tiragolumab, Nab-Paclitaxel, Carboplatin, Doxorubicin, Cyclophosphamide, Granulocyte colony-stimulating factor (G-CSF), Granulocyte-Macrophage colony-stimulating factor (GM-CSF)	I	80	Estimated Primary Completion Date: 15 March 2022	[69]
NCT03498716	Recruiting	Chemotherapy (Paclitaxel, Dose-dense Doxorubicin/Epirubicin, Cyclophosphamide)	III	2300	Estimated Primary Completion Date: 31 May 2024	[70]
NCT03197935	Active, not recruiting	Nab-Paclitaxel, Doxorubicin, Cyclophosphamide, Filgastrim, Pegfilgastrim, Placebo	III	333	In patients with early-stage TNBC, neoadjuvant treatment with atezolizumab in combination with nab-paclitaxel and anthracycline-based chemotherapy significantly improved pathological complete response rates with an acceptable safety profile.	[71]
NCT04177108	Active, not recruiting	Ipatasertib, Paclitaxel, Placebo for Atezolizuamb, Placebo for Ipatasertib	III	242	Estimated Primary Completion Date: 10 October 2025	[72]
NCT03256344	Active, not recruiting	Talimogene Laherparepvec	I	36	Estimated Study Completion Date: 25 August 2022	[73]
NCT03292172	Terminated	RO6870810	I	36	The study has stopped early and will not start again. Participants are no longer being examinated or treated.	[74]
NCT04739670	Not yet recruiting	Bevacizumab, Carboplatin, Gemcitabine	II	31	Estimated Primary Completion Date: September 2025	[75]
NCT03371017	Recruiting	Chemotherapy (Gemcitabine, Capecitabine, Carboplatin), Placebo for atezolizumab	III	572	Estimated Primary Completion Date: 31 January 2023	[76]
NCT04770272	Recruiting	Atezolizumab 840 MG in 14 ML Injection, Atezolizumab 1200 MG in 20 ML Injection, Carboplatin, Paclitaxel, Epirubicin, Cyclophosphamide, Biopsy Arm A, Biopsy Arm B, Surgery	II	458	Estimated Primary Completion Date: 1 August 2023	[77]
NCT03206203	Active, not recruiting	Carboplatin	II	106	Estimated Primary Completion Date: 30 November 2022	[78]
NCT03756298	Recruiting	Capecitabine, Capecitabine in monotherapy	II	284	Estimated Primary Completion Date: 31 January 2024	[79]
NCT02530489	Active, not recruiting	Atezolizumab + Nab-Paclitaxel	II	37	Estimated Primary Completion Date: 28 February 2022	[80]
NCT04408118	Recruiting	Paclitaxel, Bevacizumab	II	100	Estimated Primary Completion Date: October 2022	[81]
NCT03853707	Suspended	Capecitabine, Carboplatin, Ipatasertib, Paclitaxel	I, II	40	Estimated Primary Completion Date: 21 June 2023	[82]
NCT03483012	Active, not recruiting	Stereotactic radiosurgery (SRS)	II	45	Estimated Primary Completion Date: 30 September 2021	[83]
NCT03101280	Completed	Rucaparib	I	29	No Study Results Posted	[84]
NCT01898117	Recruiting	Carboplatine and Cyclophosphamide, Carboplatine and Cyclophosphamide in monotherapy, Paclitaxel, Paclitaxel in monotherapy	II	304	Estimated Primary Completion Date: December 2024	[85]
NCT03464942	Recruiting	Stereotactic Ablative Body Radiotherapy (SABR)	II	52	Estimated Study Completion Date: April 2022	[86]
NCT02883062	Active, not recruiting	Carboplatin, Lumpectomy, Mastectomy, Paclitaxel	II	72	Estimated Primary Completion Date: 1 July 2022	[87]
NCT04249167	Active, not recruiting	Cryosurgery, Nab-Paclitaxel	I	5	Estimated Primary Completion Date: 31 December 2021	[88]
NCT02322814	Completed	Cobimetynib, Paclitaxel, Nab-Paclitaxel, Placebo	II	169	Percentage of Participants with Confirmed Overall Response (OR): Cohort II (Cobimetinib, Paclitaxel, Atezolizumab): 34.4%, Cohort III (Cobimetinib, Nab-Paclitaxel, Atezolizumab): 29%, Serious Adverse Events: Cohort II: 46.88%, Cohort III: 43.33%	[89]
NCT04434040	Recruiting	Sacituzumab govitecan	II	40	Estimated Primary Completion Date: 30 December, 2023	[90]
NCT03800836	Active, not recruiting	Ipatasertib, Paclitaxel, Nab-Paclitaxel, AC (Doxorubicin and Cyclophosphamide)	I	140	Estimated Primary Completion Date: 29 October 2022	[91]
NCT03961698	Recruiting	IPI-549 (eganelisib), Nab-Palitaxel, Bevacizumab	II	90	Estimated Primary Completion Date: 1 August 2022	[92]
NCT02620280	Active, not recruiting	Carboplatin, Nab-Paclitaxel, Anthra-AC or EC (adriamycin or epirubicin and cyclophosphamide or FEC (fluorouracil, epirubicin and cyclophosphamide)	III	278	Estimated Primary Completion Date: May 2022	[93]
NCT04849364	Recruiting	Capecitabine, Talazoparib, Inavolisib,	II	197	Estimated Primary Completion Date: January 2024	[94]
NCT04639245	Recruiting	Atezolizumab, Cyclophosphamide, Fludarabine, MAGE-A1-specific T-cell Receptor-transduced Autologous T-cells, PD1 Inhibitor	I, II	18	Estimated Primary Completion Date: 1 December 2024	[95]
NCT02708680	Unknown	Etinostat, Placebo for atezolizumab	I, II	88	No Study Results Posted	[96]
NCT03915678	Recruiting	BDB001, Radiotherapy	II	247	Estimated Primary Completion Date: September 2023	[97]
NCT03424005	Recruiting	Capecitabine, Ipatasertib, SGN-LIV1A, Bevacizumab, Chemotherapy (Gemcitabine, Carboplatin or Eribulin), Selicrelumab, Tocilizumab, Nab-Paclitaxel, Sacituzumab Govitecan	I, II	280	Estimated Primary Completion Date: 3 January 2023	[98]
NCT03289962	Recruiting	Autogene cevumeran	I	770	Estimated Primary Completion Date: 1 February 2024	[99]
NCT05001347	Not yet recruiting	TJ004309	II	60	Estimated Primary Completion Date: October 2024	[100]
NCT03829501	Recruiting	KY1044 and KY1044 as a single agent	I, II	412	Estimated Primary Completion Date: May 2023	[101]
NCT02543645	Terminated	Varlilumab	I	18	The study has stopped early and will not start again. Participants are no longer being examined or treated. No Study Results Posted	[102]
NCT03170960	Recruiting	Cabozantinib	I, II	1732	Estimated Primary Completion Date: December 2021	[103]
NCT04638751	Recruiting	Immunotherapy and Chemotherapeutic agent	Observational study	4000	Estimated Primary Completion Date: December 2022	[104]
NCT03232593	Recruiting	Monotherapy	Phase 4, surveillance study	3000	Estimated Primary Completion Date: 11 January 2023	[105]
NCT03952325	Terminated	Pembrolizumab, Tesetaxel, Nivolumab	II	294	The Sponsor has discontinued the development of tesetaxel. No Study results Posted	[106]
NCT04102618	Recruiting	Trastuzumab, Pelareoreb, Letrazole	I	38	No Study Results Posted	[107]
NCT04954599	Not yet recruiting	CP-506, Carboplatin, Immune checkpoint inhibitor (including atezolizumab)	I, II	126	Estimated Primary Completion Date: September 2024	[108]
NCT05069935	Not yet recruiting	FT538, Cyclophosphamide, Fludarabine, Combination Product: Monoclonal antibody (including Atezolizumab)- Dose Escalation Combination Product: Monoclonal antibody (including Atezolizumab)—Dose Expansion	I	189	Estimated Primary Completion Date: 5 September 2023	[109]

**Table 2:** Pembrolizumab.

NCT Number	Status	Co-Treatment/Intervention	Phase	Participants	Results/Conclusions	References
NCT04683679	Recruiting	Olaparib, Radiation	II	56	Estimated Primary Completion Date: January 2025	[110]
NCT04095689	Recruiting	Docetaxel, IL-12 gene therapy, L-NMMA	II	30	Estimated Primary Completion Date: January 2024	[111]
NCT04427293	Recruiting	Lenvatinib,	I	12	Estimated Primary Completion Date: July 2026	[112]
NCT04024800	Active, not recruiting	AE37 Peptide vaccine	II	29	Estimated Primary Completion Date: 30 June 2023	[113]
NCT02977468	Recruiting	Intraoperative radiation therapy (IORT)	I	15	Estimated Primary Completion Date: 31 December 2022	[114]
NCT03362060	Active, not recruiting	PVX-410	I	20	Estimated Primary Completion Date: 31 December 2022	[115]
NCT04191135	Active, not recruiting	Olaparib, Carboplatin, Gemcitabine	II, III	1225	Estimated Primary Completion Date: 26 January 2026	[116]
NCT02768701	Active, not recruiting	Cyclophosphamide	II	40	Estimated Primary Completion Date: 15 March 2023	[117]
NCT02622074	Completed	Nab-paclitaxel, Anthracycline (doxorubicin), Cyclophosphamide, Carboplatin, Palictaxel	I	60	Combination neoadjuvant chemotherapy and pembrolizumab for high-risk, early-stage TNBC showed manageable toxicity and promising antitumor activity. In an exploratory analysis, the pCR rate showed a positive correlation with tumor PD-L1 expression and sTIL levels	[118]
NCT03720431	Active, not recruiting	TTAC-0001	I	11	Estimated Study Completion Date: 26 February 2022	[119]
NCT03121352	Active, not recruiting	Nac-paclitaxel, Carboplatin	II	30	Estimated Primary Completion Date: 6 February 2022	[120]
NCT03036488	Active, not recruiting	Carboplatin. Paclitaxel, Doxorubicin, Epirubicin, Cyclophosphamide, Granulocyte colony stimulating factor: Filgrastim or Pegfilgrastim, Placebo for pembrolizumab	III	1174	Estimated Primary Completion Date: 30 September 2025	[121]
NCT03145961	Active, not recruiting	Monotherapy	II	208	Estimated Study Completion Date: 1 December 2022	[122]
NCT02819518	Active, not recruiting	Nab-Paclitaxel, Paclitaxel, Gemcitabine, Carboplatin, Normale Saline Solution (placebo)	III	882	Pembrolizumab-chemotherapy showed a significant and clinically meaningful improvement in progression-free survival versus placebo-chemotherapy among patients with metastatic triple-negative breast cancer with CPS of 10 or more. These findings suggest a role for the addition of pembrolizumab to standard chemotherapy for the first-line treatment of metastatic triple-negative breast cancer.	[40]
NCT03567720	Recruiting	Tavokinogene telseplasmid, Immunopulse (electroporation), Nab-paclitaxel	II	65	Estimated Primary Completion Date December 2023	[123]
NCT02555657	Completed	Capecitabine, Eribulin, Gemcitabine, Vinorelbine	III	622	Pembrolizumab did not significantly improve overall survival in patients with previously treated metastatic triple-negative breast cancer versus chemotherapy.	[124]
NCT03639948	Recruiting	Carboplatin, Docetaxel, Pegfilgrastim	II	100	Estimated Primary Completion Date: November 2021	[125]
NCT03184558	Terminated	Bemcentinib	II	29	Disease Control Rate (DCR): 3.4% of participants Progression-free Survival (PFS): 13.1 weeks Overall Survival (OS): 32.0 weeks	[126]
NCT04986852	Not yet recruiting	Olinvacimab	II	36	Estimated Primary Completion Date: 28 February 2025	[127]
NCT04468061	Recruiting	Sacituzumab govitecan	II	110	Estimated Primary Completion Date: 1 June 2023	[128]
NCT02734290	Active, not recruiting	Paclitaxel, Capecitabine	I, II	29	Estimated Completion Date: May 2022	[129]
NCT03752723	Recruiting	GX-I7, Cyclophosphamide	I, II	83	Estimated Study Completion Date: December 2021	[130]
NCT02981303	Completed	Imprime PGG	II	64	No Study Results Posted	[131]
NCT03644589	Withdrawn	Cisplatin	II	0	Withdrawn (No participants enrolled).	[132]
NCT02755272	Recruiting	Carboplatin, Gemcitabine	II	87	Estimated Primary Completion Date: October 2022	[133]
NCT04373031	Recruiting	IRX 2	II	30	Estimated Primary Completion Date: June 2024	[134]
NCT02971761	Active, not recruiting	Enobosarm, Laboratory Biomarker Analysis	II	29	Results Submitted—Quality Control (QC) Review Has Not Concluded	[135]
NCT02513472	Completed	Eribulin mesylate	I, II	258	ORR 25.8% in participants with mTNBC who were never treated with systemic anticancer therapy in the metastatic setting, 21.8% in participants with mTNBC previously treated with 1 to 2 lines of systemic anticancer therapy in the metastatic setting, 23.4% in participants with mTNBC who were never treated with systemic anticancer therapy and previously treated with 1 to 2 lines of systemic anticancer therapy in the metastatic setting	[136]
NCT03310957	Recruiting	Ladiratuzumab vedotin	I, II	161	Estimated Primary Completion Date: 28 February 2022	[137]
NCT02447003	Completed	Monotherapy	II	254	Safe, durable antitumor activity in a subset of patients with previously treated mTNBC. Safe, durable antitumor activity as first-line therapy for patients with PD-L1-positive mTNBC.	[36,37,38]
NCT03599453	Active, not recruiting	Chemokine Modulation Therapy, Celecoxib, Recombinant Interferon Alfa-2b, Rintatolimod	I	8	Estimated Study Completion Date: 6 July 2022	[138]
NCT03106415	Recruiting	Binimetynib, Laboratory Biomarker Analysis	I, II	38	Estimated Primary Completion Date: 15 November 2022	[139]
NCT02730130	Active, not recruiting	Radiotherapy	II	17	Complete Response in 3 participants (17.6%), and Duration of Response: 4.5 months, Time to Response 2.8 months	[140]
NCT04634747	Not yet recruiting	PVX-410 chemotherapy	II	53	Estimated Primary Completion Date: 1 April 2023	[141]
NCT03225547	Active, not recruiting	Mifepristone	II	74	Estimated Primary Completion Date: September 2022	[142]
NCT02657889	Completed	Niraparib	I, II	122	Combination niraparib plus pembrolizumab provides promising antitumor activity in patients with advanced or metastatic TNBC, with numerically higher response rates in those with tumor BRCA mutations. The combination therapy was safe with a tolerable safety profile, warranting further investigation.	[143]
NCT03012230	Recruiting	Ruxolitinib Phosphate, Laboratory Biomarker Analysis	I	18	Estimated Primary Completion Date: 1 March 2022	[144]
NCT02411656	Recruiting	Laboratory Biomarker Analysis	II	35	Estimated Primary Completion Date: 31 December 2023	[145]
NCT01676753	Active, not recruiting	Dinaciclib,	I	32	Estimated Study Completion Date: 31 December 2022	[146]
NCT04301011	Recruiting	TBio-6517	I, II	114	Estimated Primary Completion Date: 20 August 2022	[147]
NCT04348747	Not yet recruiting	Anti-HER2/HER3 Dendritic Cell Vaccine, Celecoxib, Recombinant Interferon Alfa-2b, Rintatolimod	II	23	Estimated Primary Completion Date: 1 December 2023	[148]
NCT04879849	Recruiting	TAK-676, radiotherapy	I	46	Estimated Primary Completion Date: 18 January 2024	[149]
NCT05082259	Not yet recruiting	ASTX660	I	48	Estimated Primary Completion Date: 16 March 2026	[150]
NCT04230109	Active, not recruiting	Sacituzumab Govitecan	II	51	Estimated Primary Completion Date: 30 October 2024	[151]
NCT03197389	Completed	Monotherapy	I	54	Among patients with TNBC, administration of single dose of pembrolizumab was not correlated with PD-1 expression in patients with or without neoadjuvant chemotherapy.	[152]
NCT04443348	Recruiting	Radiation Therapy Boost, Paclitaxel, Carboplatin, Cyclophosphamide, Doxorubicin, Capecitabine	II	120	Estimated Primary Completion Date: 1 June 2023	[153]
NCT05112536	Recruiting	Pembrolizumab, Trilaciclib, Cylophosphamide, Doxorubicin, Paclitaxel, Carboplatin	II	30	Estimated Primary Completion Date: 20 August 2022	[154]
NCT03775850	Completed	EDP1503	I, II	69	No Study Results Posted	[155]
NCT03289819	Completed	Nab-paclitaxel Epirubicin Cyclophosphamide	II	53	No Study Results Posted	[156]
NCT04432857	Recruiting	AN0025	I	84	Estimated Primary Completion Date: December 2023	[157]
NCT03396445	Recruiting	MK-5890, Pemetrexed, Carboplatin, Nab-paclitaxel	I	202	Estimated Primary Completion Date: 25 October 2024	[158]
NCT01986426	Completed	LTX-315	I	80	No Study Results Posted	[159]
NCT03761914	Recruiting	Galinpepimut-S	I, II	90	Estimated Primary Completion Date: 31 January 2024	[160]
NCT03797326	Active, not recruiting	Lenvatinib	II	590	Estimated Primary Completion Date: 22 December 2023	[161]
NCT04265872	Recruiting	Bortezomib, and cisplatin injections--bortezomib followed by pembro/cis	Early phase I	20	Estimated Primary Completion Date: 1 October 2023	[162]
NCT04332653	Recruiting	Efineptakin alfa	I, II	178	Estimated Primary Completion Date: 30 June 2022	[163]
NCT04429542	Recruiting	BCA101	I	292	Estimated Primary Completion Date: 31 December 2022	[164]
NCT05082610	Not yet recruiting	HMBD-002	I	240	Estimated Primary Completion Date: October 2024	[165]
NCT02644369	Active, not recruiting	Monotherapy	II	100	No Study Results Posted	[166]
NCT05094804	Recruiting	OR2805, Nivolumab	I, II	130	Estimated Primary Completion Date: 15 April 2024	[167]
NCT05070247	Not yet recruiting	TAK-500	I	106	Estimated Primary Completion Date: 8 April 2025	[168]
NCT04725331	Recruiting	BT-001	I, II	48	Estimated Primary Completion Date: 30 November 2024	[169]
NCT03454451	Recruiting	Ciforadenant, CPI-006	I	378	Estimated Primary Completion Date: March 2022	[170]
NCT03849469	Recruiting	XmAb^®^22841	I	242	Estimated Primary Completion Date: June 2024	[171]
NCT04234113	Recruiting	SO-C101	I	96	Estimated Primary Completion Date: December 2023	[172]
NCT02178722	Completed	Epacadostat	I, II	444	Ammong patients with TNBC Safety, ORR: 11.1%	[173]
NCT05007106	Recruiting	Vibostolimab Co-Formulation, Lenvatinib, 5-Fluorouracil, Cisplatin, Paclitaxel	II	480	Estimated Primary Completion Date: 19 February 2025	[174]
NCT03621982	Recruiting	ADCT-301	I	95	Estimated Primary Completion Date: 15 November 2022	[175]
NCT04348916	Recruiting	ONCR-177	I	132	Estimated Primary Completion Date: January 2025	[176]
NCT01042379	Recruiting	Standard Therapy, AMG 386 with or without Trastuzumab, AMG 479 (Ganitumab) plus Metformin, MK-2206 with or without Trastuzumab, AMG 386 and Trastuzumab, T-DM1 and Pertuzumab, Pertuzumab and Trastuzumab, Ganetespib, ABT-888, Neratinib, PLX3397, Pembrolizumab- 4 cycle, Talazoparib plus Irinotecan, Patritumab and Trastuzumab, Pembrolizumab-8 cycle, SGN-LIV1A, Durvalumab plus Olaparib, SD-101 + Pembrolizumab, Tucatinib plus trastuzumab and pertuzumab, Cemiplimab, Cemiplimab plus REGN3767, Trilaciclib with or without trastuzumab + pertuzumab, SYD985 ([vic-]trastuzumab duocarmazine), Oral Paclitaxel + Encequidar + Dostarlimab (TSR-042) + Carboplatin with or without trastuzumab, Oral Paclitaxel + Encequidar + Dostarlimab (TSR-042) with or without trastuzumab, Amcenestrant, Amcenestrant + Abemaciclib Amcenestrant + Letrozole	II	4000	Estimated Primary Completion Date: December 2030	[177]
NCT04060342	Recruiting	GB1275D, Nab-paclitaxel and gemcitabine	I, II	242	Estimated Primary Completion Date: March 2023	[178]
NCT03366844	Recruiting	Radiotherapy	I, II	60	Estimated Primary Completion Date: 21 January 2022	[179]
NCT04148937	Active, not recruiting	LY3475070	I	150	Estimated Primary Completion Date: 20 December 2021	[180]
NCT03277352	Terminated	Epacadostat	I, II	10	The study was terminated due to emergent data from another study and unrelated to safety. Treatment-Emergent Adverse Events: 100% ORR: 30%	[181]
NCT05069935	Not yet recruiting	FT538, Cyclophosphamide, Fludarabine, Monoclonal antibody including pembrolizumab—Dose Escalation, Monoclonal antibody (including pembrolizumab)—Dose Expansion	I	189	Estimated Primary Completion Date 5 September 2023	[109]
NCT03952325	Terminated (The Sponsor has discontinued the development of tesetaxel)	Atezolizumab, Nivolumab, Tesetaxel	II	294	The study has stopped early and will not start again. Participants are no longer being examined or treated.	[106]
NCT04954599	Not yet recruiting	CP-506 Carboplatin Immune checkpoint inhibitor (including pembrolizumab)	I, II	126	Estimated Primary Completion Date: September 2024	[108]

**Table 3:** Nivolumab.

NCT Number	Status	Co-Treatment/Intervention	Phase	Participants	Results/Conclusions	References
NCT04331067	Recruiting	Paclitaxel, Carboplatin, Cabiralizumab, Tumor biopsy, Bone marrow, Blood draw	I, II	50	Estimated Primary Completion Date: 31 December 2022	[182]
NCT02393794	Active, not recruiting	Romidepsin, Cisplatin	I, II	51	Estimated Primary Completion Date: July 2022	[183]
NCT03818685	Recruiting	Ipilimumab, Capecitabine	II	114	Estimated Primary Completion Date: 1 February 2022	[184]
NCT03487666	Active, not recruiting	Capecitabine	II	45	Estimated Primary Completion Date: December 2021	[185]
NCT03414684	Active, not recruiting	Carboplatin	II	78	Estimated Primary Completion Date: 30 December 2021	[186]
NCT03316586	Completed	Cabozantinib	II	18	Results Submitted—Quality Control (QC) Review Has Not Concluded	[187]
NCT02499367	Active, not recruiting	Radiation therapy, Low dose doxorubicin, Cyclophosphamide, Cisplatin	II	84	Estimated Primary Completion Date December 2021	[188]
NCT03098550	Complted	Daratumumab	I, II	105	Among patients with TNBC: Adverse events occurred in 100% and serious adverse events in 70.7%. ORR was 4.9% and PFS was 1.22 months	[189]
NCT04159818	Recruiting	Cisplatin, Low dose doxorubicin	II	52	Estimated Primary Completion Date: 15 December 2022	[190]
NCT04142931	Recruiting	ImmunicomAIAC	I	30	Estimated Primary Completion Date: 30 December 2021	[191]
NCT02834247	Terminated	TAK-659	I	41	Insufficient efficacy of drug; no safety concern	[192]
NCT03546686	Recruiting	Ipilimumab, Core Biopsy/Cryoablation, Breast Surgery	II	80	Estimated Primary Completion Date: June 2022	[193]
NCT05094804	Recruiting	Pembrolizumab, OR2805	I, II	130	Estimated Primary Completion Date: 15 April 2024	[167]
NCT03435640	Active, not recruiting	Bempegaldesleukin, NKTR-262	I, II	64	Estimated Primary Completion Date: July 2022	[194]
NCT02637531	Active, not recruiting	IPI-549 (eganelisib)	I	219	Estimated Study Completion Date: October 2022	[195]
NCT03829436	Recruiting	Part 1 TPST-1120 Part 2 TPST-1120 + nivolumab Part 3 TPST-1120 Part 4 TPST-1120 + nivolumab	I	138	Estimated Primary Completion Date: 18 February 2022	[196]
NCT04423029	Recruiting	DF6002	I, II	380	Estimated Primary Completion Date: September 2022	[197]
NCT03667716	Recruiting	COM701 co-treatment and COM701 monotherapy,	I	140	Estimated Primary Completion Date: December 2022	[198]
NCT04561362	Recruiting	BT8009	I, II	146	Estimated Primary Completion Date: June 2023	[199]
NCT04638751	Recruiting	Immunotherapy (Including Nivolumab) Chemotherapeutic Agent	Observational	4000	Estimated Primary Completion Date: December 2022	[104]
NCT05069935	Not yet recruiting	FT538, Cyclophosphamide, Fludarabine, Combination Product: Monoclonal antibody (including Nivolumab)- Dose Escalation Combination Product: Monoclonal antibody (including Nivolumab)—Dose Expansion	I	189	Estimated Primary Completion Date: 5 September 2023	[109]
NCT03952325	Terminated	Pembrolizumab, Tesetaxel, Nivolumab	II	294	The Sponsor has discontinued the development of tesetaxel. No Study results Posted	[106]
NCT04954599	Not yet recruiting	CP-506, Carboplatin, Immune checkpoint inhibitor (including Nivolumab)	I, II	126	Estimated Primary Completion Date: September 2024	[108]

**Table 4:** Avelumab.

NCT Number	Status	Co-Treatment/Intervention	Phase	Participants	Results/Conclusions	References
NCT02926196	Not yet recruiting	Monotherapy	III	474	Estimated Primary Completion Date: December 2021	[200]
NCT04360941	Recruiting	Palbociclib	I	45	Estimated Primary Completion Date: 1 January 2024	[201]
NCT04188119	Not yet recruiting	Aspirin, Lansoprazole	II	42	Estimated Primary Completion Date: 30 August 2022	[202]
NCT03971409	Recruiting	Anti-OX40 Antibody PF-04518600, Binimetinib, Utomilumab, Liposomal Doxorubicin, Sacituzumab Govitecan	II	150	Estimated Primary Completion Date: 30 July 2023	[203]
NCT03387085	Unknown	Aldoxorubicin HCl, N-803, ETBX-011, ETBX-051, ETBX-061, GI-4000, GI-6207, GI-6301, haNK for Infusion, Bevacizumab, Capecitabine, Cisplatin, Cyclophosphamide, 5-Fluorouracil, Leucovorin, nab-Paclitaxel, SBRT	I, II	79	No study results posted	[204]
NCT03861403	Terminated	TRX518, Cyclophosphamide	I, II	10	No study results posted	[205]
NCT02630368	Recruiting	Cyclophosphamide and JX-594 dose escalation, Cyclophosphamide and JX-594, Cyclophosphamide as a single agent, Avelumab and JX-594 and Cyclophosphamide	I, II	197	Estimated Primary Completion Date: May 2023	[206]
NCT04638751	Recruiting	Immunotherapy (including avelumab), Chemotherapeutic agent	Observational	4000	Estimated Primary Completion Date: December 2022	[104]
NCT02554812	Active, not recruiting	Utomilumab, PF-04518600, PD 0360324, CMP-001	II	398	Estimated Primary Completion Date: 29 April 2022	[207]
NCT05069935	Not yet recruiting	FT538, Cyclophosphamide, Fludarabine, Combination Product: Monoclonal antibody (including Avelumab)—Dose EscalationCombination Product: Monoclonal antibody (including Avelumab)—Dose Expansion	I	189	Estimated Primary Completion Date: 5 September 2023	[109]
NCT04551885	Active, not recruiting	FT516, Fludarabine, Cyclophosphamide, Il-2	I	12	Estimated Primary Completion Date: August 2022	[208]
NCT02222922	Completed	PF-06647020 Q3W, Fluconazole, PF-06647020 Q2W, PF-06647020 combined with Avelumab	I	138	No study results for avelumab co-treatment	[209]
NCT04954599	Not yet recruiting	CP-506 Carboplatin Immune checkpoint inhibitor (including pembrolizumab)	I, II	126	Estimated Primary Completion Date: September 2024	[108]
NCT01772004	Completed	Monotherapy	I	1756	The anti-PD-L1 antibody avelumab has a safety profle that is considered generally manageable and tolerable, and showed modest clinical activity in a heavily pretreated population of patients with metastatic BC. Durable clinical beneft can be achieved with anti-PD-1/PD-L1 monotherapy in a subset of patients with metastatic BC, particularly TNBC.	[48]

**Table 5:** Cemiplimab.

NCT Number	Status	Co-Treatment/Intervention	Phase	Participants	Results/Conclusions	References
NCT04243616	Recruiting	Paclitaxel, Carboplatin (not mandatory), Doxorubicin, Cyclophosphamide	II	36	Estimated Primary Completion Date: 15 March 2022	[210]
NCT04638751	Recruiting	Immunotherapy (including cemiplimab) Chemotherapeutic Agent	Observational study	4000	Estimated Primary Completion Date: December 2022	[104]
NCT01042379	Recruiting	Standard Therapy, AMG 386 with or without Trastuzumab, AMG 479 (Ganitumab) plus Metformin, MK-2206 with or without Trastuzumab, AMG 386 and Trastuzumab, T-DM1 and Pertuzumab, Pertuzumab and Trastuzumab, Ganetespib, ABT-888, Neratinib, PLX3397, Pembrolizumab- 4 cycle, Talazoparib plus Irinotecan, Patritumab and Trastuzumab, Pembrolizumab-8 cycle, SGN-LIV1A, Durvalumab plus Olaparib, SD-101 + Pembrolizumab, Tucatinib plus trastuzumab and pertuzumab, Cemiplimab, Cemiplimab plus REGN3767, Trilaciclib with or without trastuzumab + pertuzumab, SYD985 ([vic-]trastuzumab duocarmazine), Oral Paclitaxel + Encequidar + Dostarlimab (TSR-042) + Carboplatin with or without trastuzumab, Oral Paclitaxel + Encequidar + Dostarlimab (TSR-042) with or without trastuzumab, Amcenestrant, Amcenestrant + Abemaciclib Amcenestrant + Letrozole	II	4000	Estimated Primary Completion Date: December 2030	[177]
NCT04954599	Not yet recruiting	CP-506, Carboplatin, Immune checkpoint inhibitor (including cemiplimab)	I, II	126	Estimated Primary Completion Date: September 2024	[108]

**Table 6:** Durvalumab.

NCT Number	Status	Co-Treatment/Intervention	Phase	Participants	Results/Conclusions	References
NCT03199040	Active, not recruiting	Neoantigen DNA vaccine	I	10	Estimated Study Completion Date: 3 December 2022	[211]
NCT03167619	Active, not recruiting	Olaparib	II	50	Estimated Study Completion Date: 30 June 2022	[212]
NCT02826434	Active, not recruiting	PVX-410 Hiltonol	I	22	Estimated Study Completion Date: September 2022	[213]
NCT02489448	Active, not recruiting	Nab-paclitaxel, Tremelimumab	I, II	71	Estimated Study Completion Date: December 2021	[214]
NCT02527434	Active, not recruiting	Monotherapy, Tremelimumab combination therapy	II	64	1 patient completed, 11 not completed (death 6, withdrawal by subject 3, lost to follow-up 2)	[215]
NCT03982173	Active, not recruiting	combination with tremelimumab	II	88	Estimated Study Completion Date: April 2023	[216]
NCT03616886	Recruiting	Paclitaxel Carboplatin Oleclumab (MEDI9447)	I, II	171	Estimated Study Completion Date: October 2023	[217]
NCT03356860	Recruiting	Paclitaxel Epirubicin Cyclophosphamide	I, II	57	Estimated Study Completion Date: January 2022	[218]
NCT03742102	Recruiting	Capivasertib Oleclumab Paclitaxel Trastuzumab deruxtecan Datopotamab deruxtecan	I, II	200	Estimated Study Completion Date: 13 February 2023	[219]
NCT04176848	Recruiting	CFI-400945	II	28	Estimated Study Completion Date: 31 December 2022	[220]
NCT03801369	Recruiting	Olaparib	II	28	Estimated Study Completion Date: 31 December 2026	[221]
NCT03606967	Recruiting	Carboplatin Gemcitabine Hydrochloride Nab-paclitaxel Personalized Synthetic Long Peptide Vaccine Poly ICLC Tremelimumab	II	70	Estimated Study Completion Date: 31 December 2021	[222]
NCT03740893	Recruiting	AZD6738 Olaparib	II	81	Estimated Study Completion Date: December 2025	[223]
NCT03739931	Recruiting	mRNA-2752	I	264	Estimated Study Completion Date: 30 January 2023	[224]
NCT04504669	Recruiting	AZD8701	I	123	Estimated Study Completion Date: 7 September 2023	[225]
NCT03983954	Recruiting	Naptumomab estafenatox (ABR-217620; NAP) Obinutuzumab pretreatment (Gazyva^®^)	I	50	Estimated Study Completion Date: 28 July 2022	[226]
NCT04638751	Recruiting	Chemotherapeutic Agent	Prospective study	4000	Estimated Study Completion Date: December 2024	[104]
NCT01042379	Recruiting	In combination with Olaparib	II	4000	Estimated Study Completion Date: December 2031	[177]
NCT04556773	Recruiting	In combination with Trastuzumab deruxtecan and Paclitaxel	I	185	Estimated Study Completion Date: December 2031	[227]
NCT04954599	Not yet recruiting	CP-506 Carboplatin Immune checkpoint inhibitor	I, II	126	Estimated Study Completion Date: October 2024	[108]
NCT03544125	Completed	Olaparib	I	3	No Study Results Posted	[228]
NCT02685059	Completed	Placebo, Nab-Paclitaxel, Epirubicin, Cyclophosphamide	II	174	The addition of durvalumab to anthracycline-/taxane-based NACT increases pCR rate particularly in patients treated with durvalumab alone before start of chemotherapy.	[43]
NCT02628132	Completed	Paclitaxel	I, II	22	Safety of therapy. The confirmed objective response rate (ORR) was observed in five patients with a median duration of 10.0 months. Median Progression-free survival (PFS) and overall survival (OS) were 5 and 20.7 months, respectively.	[229]
NCT02658214	Completed	Gemcitabine + carboplatin, Nab-paclitaxel (paclitaxel-albumin) + carboplatin, Tremelimumab,	I	32	No Study Results Posted	[230]
NCT03872505	Withdrawn	Radiation Therapy, Carboplatin, Paclitaxel	II	140	Lack of funding	[231]

**Table 7:** Ipilimumab.

NCT Number	Status	Co-Treatment/Intervention	Phase	Participants	Results/Conclusions	References
NCT03818685	Recruiting	Radiotherapy and Capecitabine Nivolumab	II	114	Estimated Study Completion Date: 1 December 2022	[184]
NCT03546686	Recruiting	Nivolumab Core biopsy and cryoablation	II	80	Estimated Study Completion Date: 1 December 2024	[193]
NCT04638751	Recruiting	Chemotherapeutic Agent	Obser-vational study	4000	Estimated Study Completion Date: December 2024	[104]
NCT03752398	Recruiting	XmAb^®^23104	I	234	Estimated Study Completion Date: December 2024	[232]
NCT03606967	Recruiting	Nab-Paclitaxel + Durvalumab (MEDI4736) + Tremelimumab + Neoantigen Vaccine vs. Nab-Paclitaxel + Durvalumab + Tremelimumab	II	70	No Study Results Posted	[222]
NCT04954599	Not yet recruiting	CP-506 Carboplatin Immune checkpoint inhibitor	I, II	126	No Study Results Posted	[108]
NCT04434560	Terminated	Nivolumab	II	1	Poor enrollment	[233]
NCT02983045	Active, not recruiting	Combination of NKTR-214 + nivolumab	I, II	557	Estimated Primary Completion Date November 2021	[234]
NCT03126110	Active, not recruiting	INCAGN01876	I, II	145	No Study Results Posted	[235]
NCT03241173	Completed	INCAGN01949	I, II	52	No Results Posted	[236]
NCT04879888	Completed	Peptide pulsed Dendritic cell	I	9	Restoring the responsiveness of T-cells by increasing the frequency and activation in peripheral blood of tumor specific T-cells present in the tumor	[237,238]

**Table 8:** Tremelimumab.

NCT Number	Status	Co-Treatment/Intervention	Phase	Participants	Results/Conclusions	References
NCT02527434	Active, not recruiting	Monotherapy Combination therapy with MEDI4736	II	12	1 patient completed, 11 not completed (death 6, withdrawal by subject 3, lost to follow-up 2)	[215]
NCT03982173	Active, not recruiting	Durvalumab	II	88	Estimated Study Completion Date April 2023	[216]
NCT02489448	Active, not recruiting	Nab-Paclitaxel, Durvalumab (MEDI4736)	I, II	71	Estimated Study Completion Date December 2021	[214]
NCT03606967	Recruiting	Carboplatin Durvalumab Gemcitabine Hydrochloride Nab-paclitaxel Personalized Synthetic Long Peptide Vaccine Poly ICLC	II	70	No Study Results Posted	[222]
NCT02658214	Completed	Platinum Durvalumab	I	32	No Study Results Posted	[230]
NCT03674827	Completed	A vaccine-based immunotherapy regimen (VBIR-2) (PF-06936308), Sasanlimab	I	36	No Study Results Posted	[239]
